# Pain Management of Acute and Chronic Postoperative Pain

**DOI:** 10.7759/cureus.23999

**Published:** 2022-04-09

**Authors:** Yusuke Ishida, Toshio Okada, Takayuki Kobayashi, Kaori Funatsu, Hiroyuki Uchino

**Affiliations:** 1 Anesthesiology, Tokyo Medical University Hospital, Tokyo, JPN

**Keywords:** multimodal analgesia, postsurgical analgesia, biological factors, psychological factors, chronic postsurgical pain

## Abstract

Inadequate management of acute postoperative pain is associated with effects related to both physiological and psychological function. Postoperative pain increases the risk of perioperative complications, so postoperative pain should be prevented. Postoperative pain management by sufficient analgesia is important while considering the use of various kinds of analgesics. Insufficient management of postoperative pain may lead to chronic postsurgical pain (CPSP). It is suggested that CPSP is dependent not only upon biological factors but also upon psychological factors, including the type of surgery, age, physical health, mental health, and preoperative pain. As CPSP is a severe complication that may prolong hospitalization and interferes with activities of daily living (ADL) and quality of life (QoL), its prevention of development is paramount. Therefore, in order to prevent the onset of CPSP, it is necessary to craft analgesic management to prevent CPSP during the perioperative period.

## Introduction and background

The prevention of postsurgical pain is one of the major key points of anesthetic management. Depending on the case, the use of intravenous patient-controlled analgesia (IV-PCA) and the combined administration of epidural anesthesia are considered for postsurgical analgesia. Recently, the combined use of regional anesthesia for neural blockade (such as brachial plexus block and transversus abdominis plane block) is also performed for postsurgical analgesia. Typical postsurgical analgesia methods are using opioids such as fentanyl from the time of operation and considering the use of nonsteroidal anti-inflammatory drugs (NSAIDs) and acetaminophen after surgery. In certain situations, direct local anesthesia for the wound is also carried out. In this way, we are examining postoperative analgesia using various methods and will consider its importance.

## Review

Pain mechanism and harmful effects of pain

It is known that the mechanism of inducing pain, especially pain during and after surgery, involves the activation and sensitization of the nociceptor due to surgical stress [1]. Furthermore, it is suggested that humoral factors, such as prostaglandin and cytokines that function locally (at the surgery site) and systemically, decrease tissue pH and partial pressure of oxygen (pO2), enhance the reaction of nociceptive neurons of the central nervous system (CNS), and facilitate the spontaneous excitation of neurons due to peripheral nerve injury; they are associated with the mechanism to induce pain during and after an operation [2]. Inadequate management of acute postoperative pain is associated with effects related to both physiological and psychological function [3-7]. The effects of postsurgical pain on the respiratory system include a decrease in lung capacity, functional residual capacity (FRC), tidal volume, hypertonia of the abdominal muscles, and a decrease in diaphragm function. Furthermore, the fear of pain may restrain the patient from coughing and taking deep breaths, which, in turn, may induce atelectasis and accumulation of secreted products [8]. These factors can become a cause of hypoxemia. The effects on the circulatory system include tachycardia and an increase in blood pressure due to excitation of the sympathetic nervous system. This causes an increase in oxygen consumption, which consequently causes a disruption of the balance between oxygen supply and demand. These effects raise the possibility of complications such as myocardial ischemia and myocardial infarction to occur. Prolonged bed rest due to persistent postsurgical pain may also cause the development of deep-vein thrombosis (DVT). The development of blood clots increases the risk of pulmonary thrombosis, which can become fatal. Effects on the digestive system include the suppression of intestinal movement, which can cause postsurgical ileus. Voiding dysfunction may also occur. Effects on the endocrine system include the excitation of the sympathetic nervous system, which prompts the discharge of catecholamine and catabolic hormones. This causes an increase in metabolism and oxygen consumption. Effects on the mental aspect of the patient include the overuse of analgesic drugs due to anxiety and fear of postsurgical pain. Prolonged pain may also cause a sense of distrust toward medicine. It is known that the risk of the above-mentioned complications increases as postsurgical pain becomes stronger. The development of these complications can also cause prolonged hospitalization and an increase in medical expenses. Therefore, sufficient analgesic management of postsurgical pain is important to prevent the delay of postsurgical recovery.

**Figure 1:**
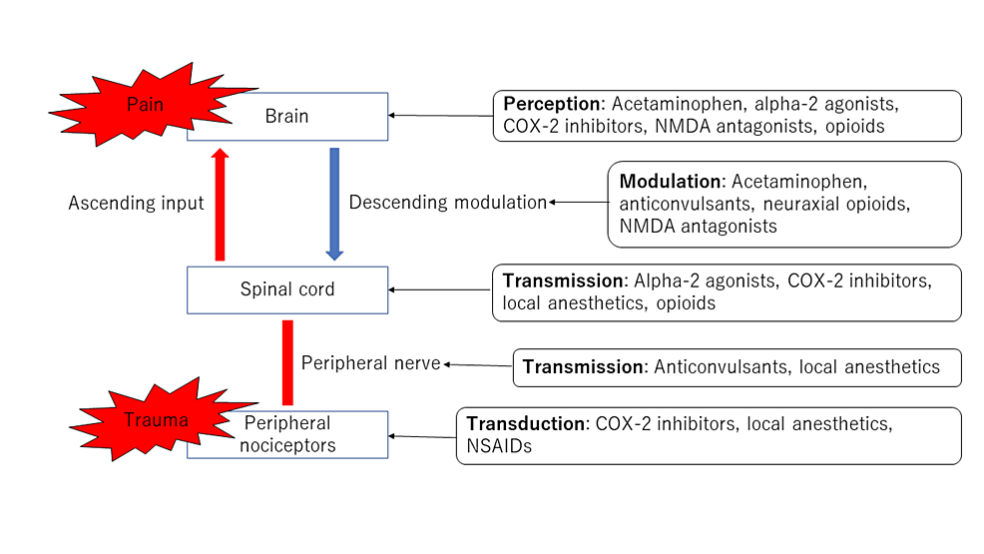
Multimodal analgesia for postoperative acute pain COX: cyclooxygenase NMDA: N-methyl-D-aspartic acid NSAIDs: nonsteroidal anti-inflammatory drugs

A guideline for postsurgical pain exists, created in the US in 2016, which includes the prevention of such pain. In this guideline, multimodal analgesia is recommended for the prevention of postsurgical pain (Figure 1) [9-10]. There are reports suggesting that multimodal analgesia increases the analgesic effect with a reduction of the total amount of opioid usage [11-12]. According to the guideline, it is recommended to administrate opioids orally rather than intravenously whenever possible for the prevention of postsurgical pain. Also, opioid administration before surgery is not recommended, and if oral administration is not possible, administration by patient-controlled analgesia (PCA) is recommended. Furthermore, monitoring of the patient is necessary when administrating opioids, as adverse events concerning analgesia and respiratory function may occur. Concerning non-steroidal anti-inflammatory drugs (NSAIDs) and acetaminophen, multimodal use is recommended. NSAIDs cause analgesic effects by inhibiting cyclooxygenase, an enzyme responsible for producing prostaglandin, a pain-inducing eicosanoid. On the other hand, acetaminophen shows analgesic effects by inhibiting the ascending pain pathway at the central level and by activating the descending pain inhibitory system mediated by serotonin [13]. Due to the differing mechanisms of analgesia, it is known that the combined administration of NSAIDs and acetaminophen increases the analgesic effect [14-15]. However, both drugs have risks of developing complications. NSAIDs are known to be associated with increasing the risk of developing gastrointestinal bleeding, ulcers, cardiovascular events, and renal dysfunction. The over-administration of acetaminophen increases the risk of hepatic dysfunction. Concerning pregabalin and gabapentin, there are reports suggesting that by co-administrating these two drugs during the perioperative period, the amount of opioid usage after the operation is reduced, and postsurgical pain also reduces [16-18]. The guideline also strongly recommends the use of the two drugs as part of multimodal analgesia.

Furthermore, in a recent randomized controlled trial (RCT) study that compared the degree and complications of acute postsurgical pain, where 122 patients performing craniotomy were divided randomly into a gabapentin-administered group and a placebo group, a significant decrease in acute postsurgical pain score within 24 hours were seen in the gabapentin-administered group [19]. The rate of developing postoperative nausea and vomiting (PONV) was also significantly lower for the gabapentin-administered group. It is also reported that the administration of dexmedetomidine, an analgesic drug that inhibits the pain pathway by acting on the α2A receptor of the spinal cord reduces postsurgical pain and thus becomes an option for the postsurgical analgesia method [20]. Analgesia by regional anesthesia, such as epidural anesthesia, is also strongly recommended by the guideline. Studies have shown that, compared to the systemic administration of opioids, the combined use of epidural anesthesia reduces the amount of analgesia used for postsurgical rescue [21] and lowers the postsurgical death rate [22]. Epidural anesthesia reduces the development of postsurgical complications, such as deep venous thrombosis (DVT), pneumonia, atelectasis, respiratory suppression, atrial fibrillation (AF), and PONV [23], and it is suggested that performing regional anesthesia whenever possible would give benign consequences concerning postsurgical analgesia.

Further, in recent years, the risk of administration of opioids has been reported. In 2017, among 70,237 drug overdose deaths, 47,600 (67.8%) involved opioids [24]. Therefore, there is a need for an analgesic method that does not use opioids, and it seems that the focus will be on postoperative analgesia with regional anesthesia in the future.

Chronic postsurgical pain (CPSP)

Insufficient analgesic control after an operation not only raises the risk of developing the acute complications mentioned above but also raises the risk of developing CPSP. Recently, CPSP is garnering much attention, and in 2017, it was decided to post a section dedicated to CPSP in the 11th revision of the International Classification of Diseases (ICD-11) [25]. The definition is described in Table 1 [26-27]. It is said that approximately 10-20% of postsurgical patients develop this complication, and thus it is something that cannot be ignored. Furthermore, approximately 1% of CPSP is treatment resistive. From a large-scale observational study, it is reported that 2.2% of patients were suspected of developing severe CPSP one year after the surgery [28]. Also, it is reported that the incidence rate and the severity of CPSP differ depending on the site of surgery.

**Table 1:** Definition of CPSP CPSP: chronic postsurgical pain

Definition of CPSP	
The pain develops after a surgical procedure or increases in intensity after the surgical procedure.	
The pain should be of at least 3-6 months’ duration and significantly affect the HR-QOL.	
The pain is either a continuation of acute post-surgery pain or develops after an asymptomatic period.	
The pain is either localized to the surgical field, projected to the innervation territory of a nerve situated in the surgical field, or referred to as a dermatome (after surgery in deep somatic or visceral tissues).	
Other causes of the pain should be excluded, e.g. infection or continuing malignancy in cancer surgery.	

**Figure 2:**
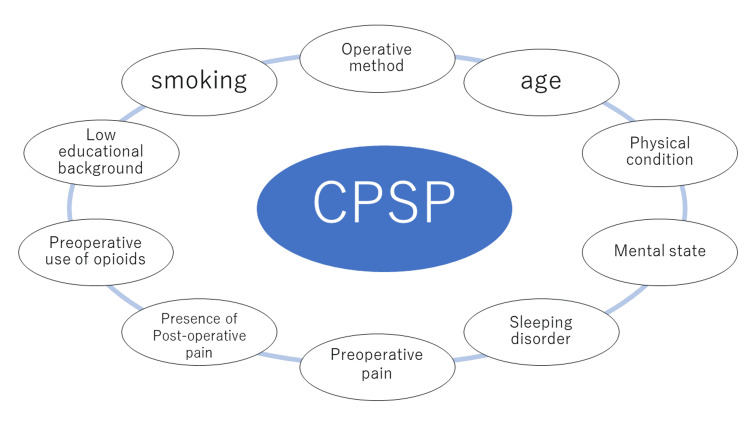
Risk factors of CPSP CPSP: chronic postsurgical pain

Montes et al. reported that the type of surgery, age of the patient, physical condition, mental condition, and presurgical pain are associated with the type of patients that are more likely to develop CPSP [29]. Concerning the type of surgery, 13.6% of patients who underwent radical surgery for hernia, 11.8% of patients who underwent a vaginal hysterectomy, 25.1% of patients who underwent an abdominal hysterectomy, and 37.6% of patients who underwent open chest surgery developed CPSP, and the incidence rate was significantly high for those who underwent open chest surgery. Detailed risk factors are shown in Figure 2 [28-31]. It is suggested from various studies that psychological factors are also associated with CPSP [32]. Studies have shown that the patient’s education level is also associated with CPSP, where patients who graduated high school had a lower incidence rate than those who did not [29].

Prevention of CPSP

Table 2 shows the summary of recent data concerning the prevention of CPSP. A study done by Bouman et al., in which they evaluated the incidence rate of CPSP of patients six months after open abdominal surgery, found that postsurgical epidermal anesthesia was associated with a reduction in the incidence rate of CPSP after open abdominal surgery [33].

**Table 2:** Treatment expected to be effective in the prevention of CPSP CPSP: chronic postsurgical pain

Treatment expected to be effective in the prevention of CPSP
Use of local or regional anesthesia
Administration of ketamine
Gabapentin, pregabalin internal use
Administration of lidocaine
Psychological approach
Rehabilitation

The analgesic mechanism of ketamine, an N-Methyl-D-aspartic acid (NMDA) receptor antagonist, is thought to be associated with the blockage of NMDA receptors in secondary neurons of the dorsal horn pain pathway and plays an important role in the enhancement of the spinal cord and cerebral cortex, which are factors of developing chronic pains. Meta-analysis of ketamine administration suggests that the incidence rate of the ketamine administration group was significantly reduced in statistical terms [34]. However, most of the studies analyzed were small-sized investigations. Thus, there is a possibility of over-evaluating the effects of the treatment, and it is necessary to be cautious when discussing the preventive effects of ketamine for CPSP. Currently, a university in Melbourne, Australia, is performing a large-scale randomized controlled trial (RCT) (the ROCKet trial) study concerning the preventive effects of ketamine for CPSP [35]. Although there is controversy concerning pregabalin and gabapentin, the most prescribed drugs for neuropathic pain, some meta-analyses report that they are effective for the prevention of CPSP [36]. Furthermore, Koh et al. reported that duloxetine, another first-choice drug, significantly reduced the degree of postsurgical pain after 12 weeks in patients who underwent artificial knee joint replacement when compared with the control group [37]. Duloxetine may be effective in patients with diminished descending pain inhibitory system. Concerning the effect of lidocaine on the inhibition of CPSP, a meta-analysis of 6 trials reported that the administration of lidocaine during the perioperative period significantly reduced the incidence rate of CPSP [38]. There are reports stating that psychological factors are also risk factors for CPSP [32,39-40], and it is suggested that psychological interventions, such as cognitive-behavioral therapy, may prevent the development of CPSP.

## Conclusions

Poor analgesic management after surgery not only increases acute complications but also raises the possibility of developing CPSP. As CPSP is a perioperative complication that prolongs hospitalization and interferes with ADLs and QoL, its prevention is important. It is also critical to mitigate the risks that are currently known to prevent the development of CPSP. It is necessary to recognize the risks of CPSP before surgery and to make sure preventive analgesics are performed after surgery. For patients at high risk, it is necessary to consider analgesic management in which CPSP would not develop prior to surgery and afterward. Careful perioperative interventions can improve a patient's prognosis and facilitate postsurgical pain control.
